# Cultivated Land Input Behavior of Different Types of Rural Households and Its Impact on Cultivated Land-Use Efficiency: A Case Study of the Yimeng Mountain Area, China

**DOI:** 10.3390/ijerph192214870

**Published:** 2022-11-11

**Authors:** Yuanhe Yu, Jinkuo Lin, Peixiang Zhou, Shuwei Zheng, Zijun Li

**Affiliations:** 1 College of Geography and Environment, Shandong Normal University, Jinan 250358, China; 2 School of Environment and Natural Resources, Renmin University of China, Beijing 100872, China; 3 School of Information and Electrical Engineering, Shandong Jianzhu University, Jinan 250101, China

**Keywords:** cultivated land input behavior, cultivated land-use efficiency, different types of rural households, data envelopment analysis, Tobit regression model, Yimeng Mountain area

## Abstract

Analyzing cultivated land input behavior (CLIB) at the scale of rural households links with cultivated land-use efficiency (CLUE), this study examined the Yimeng Mountain area in northern China, supported by field survey data from 737 rural households. This research systematically analyzed the characteristics of CLIB of different types of rural households, measured the CLUE of different types of rural households by using a data envelopment analysis (DEA) model, and explored the influence of CLIB on CLUE based on the Tobit regression model. The results show (1) significant differences in the characteristics of the CLIB of different types of rural households in the Yimeng Mountain area. Among them, the highest land, labor, and capital inputs were I part-time rural households (*I PTRH*), followed by full-time rural households (*FTRH*). In contrast, II part-time rural households (*II PTRH*) and non-agricultural rural households (*NARH*) had higher levels of non-agricultural employment; however, their input levels gradually declined. (2) The CLUE of the sample rural households was generally low and had considerable potential for improvement. Regarding the types of rural households, as the degree of part-time employment increased, the CLUE showed an inverted U-shaped trend of first increased and then decreased, namely, *I PTRH* > *FTRH* > *II PTRH* > *NARH*. This finding indicates that appropriate part-time employment could help to promote investment in agricultural production and improve the CLUE. (3) The CLIB of rural households had significant effects on CLUE; the literacy of the agricultural labor force, yield-increasing input per unit area, per capita household income, share of agricultural income, operation scale of cultivated land, effective irrigation rate of cultivated land, and soil and water conservation rate of cultivated land had positive effects on improving CLUE. Even so, there was still significant heterogeneity in the degree of influence of different rural household types. The study concluded with some policy recommendations from the perspective of different rural household types to provide references for optimizing farming inputs and improving CLUE.

## 1. Introduction

Cultivated land is the most fundamental factor of agricultural production, the basis for the survival and development of human society, and the guarantee of global food and ecological security [1]. Whether cultivated land can be used effectively is a matter of national food security and social stability; however, in a period of rapid economic and social development, especially in developing countries, cultivated land resources are under serious threat [2]. The world’s cultivated land resources per capita decreased from 0.41 hectares in 1960 to 0.21 hectares in 2019 [3]. Given the combined effects of locust invasions and the COVID-19 pandemic, since 2020, the food crisis has become a global focus and a challenge for sustainable development. China is the largest developing country worldwide, using only 7.5% of the world’s land to feed 22% of its population [4]. Although China’s self-sufficiency rate for wheat and rice has reached 98% for many years, according to data published by the FAO in 2021, China’s food self-sufficiency rate is less than 80%. Among them, the self-sufficiency rate of edible vegetable oil and soybean is relatively low, 33% and 17%, respectively, and it still needs to import agricultural products from abroad. With the accelerated urbanization and industrialization in China, the quantity and quality of cultivated land have been declining. According to the Bulletin of the Main Data of the Third National Land Survey released by the Ministry of Natural Resources in 2021, China’s cultivated land has been reduced to 127,861,900 hectares (1918 million mu), and the cultivated land per capita is only 0.09 hectares (1.37 mu). It is necessary to serve the development of agriculture and consider the effect of intensification of cultivated land and rational allocation of resources to maximize the potential value of cultivated land [5]. In this context, reasonably adjusting cultivated land inputs and improving cultivated land-use efficiency (CLUE) has become the most effective means to ensure national food security and a significant challenge for implementing a rural revitalization strategy.

The CLUE is a reflection of the degree of comprehensive utilization of cultivated land resources within a certain period and geographical space, expressed as the interrelationship between the regional cultivated land resources and the inputs of production factors, such as capital, labor, the technology they carry, and the outputs of social and economic benefits [6]. Researchers have studied CLUE using different methods and perspectives, mainly focusing on the measurement, regional differences, spatial and temporal variations, and influencing factors among which measuring and analyzing CLUE’s influencing factors are the core of the research. Some studies use weighting methods and construct indicator systems to assess CLUE [7]; however, this evaluation method relies too much on the subjectivity of the evaluator. Some studies use parametric or nonparametric methods to evaluate CLUE from the input–output perspective, such as Stochastic Frontier Function (SFA) [8] and Data Envelopment Analysis (DEA) [9]. Since the DEA model can simultaneously handle multiple inputs and outputs without weighting assumptions, it does not require dimensionless data processing and prevents model setting errors [10]. Therefore, this method has been widely used to assess CLUE in different regions worldwide [11,12]; however, the findings vary widely depending on the study area, research time, and data [13,14,15,16,17]. Researchers have found that natural conditions [18], cultivated land resource endowment [19], regional economic development level [11], and agricultural transfer population [20] at the macro scale have significant effects on CLUE, while at the micro-scale of rural households, farmers’ age [21], farmers’ education level [22], farmers’ skills training [23], land transfer [24], and cultivated land size [25,26] are essential factors influencing CLUE. In addition, land use inputs are also an important factor affecting CLUE. If chemical fertilizer, pesticide, agricultural film and other capital elements are input too little, it tends to lead to lower CLUE; if they are input too much, it will cause environmental problems, such as water pollution, soil pollution and air pollution, which is not conducive to the sustainable development of agriculture [27,28,29]. However, these studies are mainly based on different administrative scales, such as countries, provinces, or counties [30,31]. They lack consideration of rural households’ input behavior at the micro-scale and mainly focus on external influences on CLUE, with less in-depth analysis of their effects from the perspective of cultivated land input factors.

Rural households are the fundamental subjects of economic activities and decision-making units in the economic organization of farming areas, especially in the critical period of transformation from traditional to modern agriculture in rural China [32]. The individual behavior and livelihood strategies of rural households profoundly change the internal elements, structures, and functions of agricultural production systems. Therefore, CLUE analysis at the rural household scale is linked to the decision-making of cultivated land input behavior (CLIB) in rural households [33]. Since its reform and opening up, China has experienced the world’s largest and fastest urbanization process. Similar to other countries experienced a large number of rural labors transfer to cities in the process of industrialization and urbanization in the world, such as the United States, Japan, India, Brazil and so on, many young and strong laborers with competitive employment in China tend to move to cities, leading to a rural labor shortages [34,35,36,37]. The National Bureau of Statistics released the National Migrant Worker Monitoring Survey Report in 2019, indicating that the total number of migrant workers nationwide reached 290.77 million. The transfer of rural labor to non-agricultural employment changed the income structure of rural households [38], resulting in the gradual deepening of rural households’ part-time employment. Along with the rapid advancement of the market economy, part-time employment has become an important way for rural households to maintain their livelihoods and improve their welfare [39]. From a rational perspective, rural households working outside the home and agricultural production and operation are optimal decisions under the utility maximization criterion. Therefore, the change in production and operation patterns brought by rural households’ part-time employment can inevitably significantly impact cultivated land input decisions and, thus, CLUE [40]; however, the existing research still needs improvement. First, the indicators for measuring part-time employment are relatively single, and there is a lack of examining the impact of the difference in household income structure, i.e., different degrees of part-time employment, on CLUE. Second, little consideration has been given to the different ways of allocating land, capital, and labor resources caused by differences in rural households’ business objectives, which may lead to differences in CLUE. So far, few studies have evaluated and analyzed the relationship between CLIB of different types of rural households and CLUE; however, the study is crucial to the intensive and efficient use of cultivated land resources and improving the agricultural production efficiency in rural households.

The Yimeng Mountain area is a typical soil and stone mountainous area in northern China, with undulating terrain and a fragile ecosystem, but the population density is 744 people/km^2^. The shortage, low quality of cultivated land per capita, and poor agricultural infrastructure makes the relationship between humans and land increasingly tense. Furthermore, unreasonable land-use will likely cause surface pollution and soil erosion, affecting mountainous areas’ ecological and environmental security. At present, research has not examined the relationship between rural households’ CLIB and CLUE in the Yimeng Mountain area at the microscopic scale. Therefore, this study took the Yimeng Mountain area as the research object and selected six typical counties (Yiyuan, Yishui, Mengyin, Pingyi, Yinan, and Fei) as the sample areas. The study then systematically analyzed the differences in the characteristics of CLIB of different types of rural households based on the field survey data of 737 rural households, used the DEA model to measure CLUE of different types of rural households, and the Tobit model to explore the impact of rural households’ CLIB on CLUE. Finally, corresponding policy recommendations were proposed to provide empirical support for optimizing rural households’ CLIB, guiding rural households to use cultivated land reasonably and efficiently, and promoting sustainable development of agriculture in mountainous areas.

The remainder of this study is structured as follows. Section 2 presents an overview of the Yimeng Mountain area, data sources, and research methods. Section 3 shows the results of the analysis of rural households’ CLIB and CLUE, as well as an analysis of their effects on CLUE from the perspective of cultivated land input factors. Section 4 discusses and compares the empirical results, and Section 5 concludes and proposes relevant solutions or countermeasures.

## 2. Materials and Methods

### 2.1. Study Area

**Figure 1:**
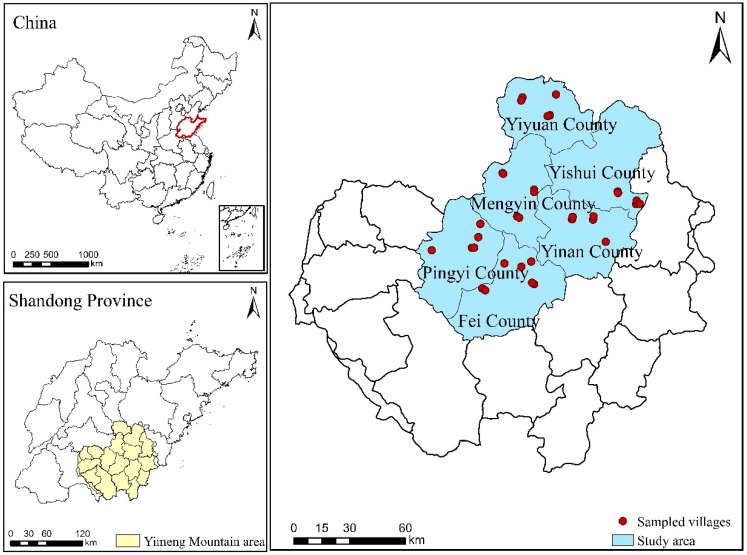
Location of the study area.

The Yimeng Mountain area is located between latitude 34°23′–36°18′ N and longitude 116°40′–119°11′ E. The administrative regions under its jurisdiction include all of counties of Mengyin, Pingyi, Fei, Yishui, Yinan, and Yiyuan, and some districts and counties in the cities of Linyi, Rizhao, Weifang, Jining, and Taian. The total area is approximately 28,804.5 km^2^ (Figure 1). The landform types are complex and diverse, with plains, hills, and mountains, each accounting for approximately 1/3 of the total study area. The terrain is high in the northwest and low in the southeast. The climate type belongs to the warm temperate continental monsoon climate, with an annual average temperature of 13–14.3 °C, annual average rainfall of 815 mm, and precipitation concentrated in summer and autumn. There are many soil types, including brown soil, cinnamon soil, and skeleton soil, and the zonal vegetation is warm temperate deciduous broad-leaved forest. Cropland (growing grain crops, oil crops, vegetables) and garden land (orchards growing peaches and apples, etc.) dominate the land-use types, and the proportion of cropland and garden land exceeds 60% of the total study area. Agricultural production occupies a large proportion of economic development; however, the level of agricultural development is relatively backward due to the poor soil and significant area of sloping cultivated land. The counties of Mengyin, Pingyi, Fei, Yishui, Yinan, and Yiyuan are the core components of the Yimeng Mountain area; all are large mountainous agricultural counties with mountains and hills accounting for a significant proportion where agriculture plays a dominant role in economic development. The land-use types in the above six counties are mainly cropland and garden land, with a wide area of sloping cultivated land. The crops mainly include wheat, maize, peanuts, and sweet potatoes, and forest and fruit crops mainly include peaches, apples, hawthorn, and chestnuts. The land-use patterns are basically the same as the overall situation in the Yimeng Mountain area. At the same time, agricultural labor emigration is more common in the six counties, and rural households’ part-time employment behavior is prominent, thus deriving different types of rural households, which is more representative of studying CLIB and CLUE in different types of rural households. The cultivated land in this study mainly refers to land where crops (grain crops, oil crops, vegetables, etc.) and perennial crops (fruit trees, chestnuts, walnuts, etc.) are grown, mainly cropland and orchards.

### 2.2. Data Sources

This study acquired data mainly from the questionnaire survey of rural households conducted by the research group from September to October 2018. The main contents of the questionnaire included five aspects: the basic characteristics of rural households, the input and output of cultivated land, the income and expenditure of rural households, the transfer of cultivated land, and the willingness of rural households to plant. In each county, 2–3 townships were selected, 2–3 villages were randomly selected in each township, 20–25 rural households were randomly selected in each village; questionnaires were conducted through interviews and seminars, etc. A total of 36 administrative villages in 18 townships were selected, 800 questionnaires were distributed, and 737 valid questionnaires (113 in Pingyi County, 139 in Fei County, 141 in Mengyin County, 111 in Yinan County, 109 in Yishui County, and 124 in Yiyuan County) were finally obtained, with an effective rate of 92.1%.

### 2.3. Methods

#### 2.3.1. Division of Rural Household Type

Currently, rural households are differentiated on various bases, and most studies neglected non-labor income other than farm and non-farm income of rural households. Some rural households have non-labor income from state policy subsidies, regional welfare subsidies, pensions, retirement wages, and child support, in addition to agricultural and non-agricultural income. Considering the actual situation of rural areas in the Yimeng Mountain area and drawing on the results of existing studies [40,41,42], this study used the rural households’ main livelihoods, the allocation of agricultural and sideline products, and the main sources and structures of household income as criteria for classifying rural households. The classification areas include full-time rural households (*FTRH*), I part-time rural households (*I PTRH*), II part-time rural households (*II PTRH*), and non-agricultural rural households (*NARH*). Table 1 presents the specific division method.

#### 2.3.2. Data Envelopment Analysis

DEA is a nonparametric technical efficiency evaluation method that can evaluate the relative effectiveness of “departments” or “units”—called decision-making units (DMUs)—with multiple inputs and outputs [4,17]. This study considers each rural household a DMU, and the relative efficiency of DMU is measured by constructing the optimal production frontier surface using DEA. If it falls on the production frontier surface, the efficiency value is 1, and the input–output is most efficient at this time. If it does not fall on the optimal frontier surface, it is inefficient, and its efficiency value is between 0 and 1. Currently, the most commonly used DEA models are the constant returns to scale (CRS) and variable returns to scale (VRS) models. The CRS model assumes that the payoff of scale is constant and measures comprehensive technical efficiency (TE), while the VRS model extends the application of the CRS model and assumes that the payoff of scale is variable and measures the pure technical efficiency (PTE) and scale efficiency (SE). The comprehensive technical efficiency (TE) can be decomposed into the product of pure technical efficiency (PTE) and scale efficiency (SE), i.e., TE = PTE × SE. Based on the measurement results of SE, it is possible to further analyze and judge the current scale payoff range of different types of rural households and adjust the production scale based on this, thereby promoting the development of CLUE in the optimal direction. The CRS model of CLUE constructed in this study is as follows:

(1) $$\left\{\begin{array}{l} Min[\theta -\varepsilon (e^{T}s^{+}+{\hat{e}}^{T}s^{-})]\\ s.t.\sum_{j=1}^{n}X_{jm}\lambda_{j}+s^{-}=\theta X_{0}\\ \sum_{j=1}^{n}Y_{jr}\lambda_{j}-s^{+}=Y_{0}\\ \lambda_{j}\geq 0,s^{-}\geq 0,s^{+}\geq 0 \end{array}\right.$$

where the CRS model treats each rural household as a DMU (*j* = 1, 2, ⋯, *n*), each rural household has *m* input and *r* output variables. *X_jm_* is the total amount of the *m_th_* input of the *j_th_* rural household, and *Y_jr_* is the total amount of the *r_th_* output of the *j_th_* rural household. *λ_j_* is the weight variable. *θ* (0 < *θ* ≤ 1) denotes the comprehensive efficiency index of cultivated land input and output. The closer *θ* is to 1, the higher the comprehensive CLUE, and *θ* = 1 is the optimal comprehensive efficiency. *s^+^* is the residual variable, *s^−^* is the relaxation variable, and *ε* is the non-Archimedean infinitesimal. *e^T^* = (1, 1, ⋯) ϵ *E^r^* and *ê^T^* = (1, 1, ⋯) ϵ *E^m^* are both unit space vectors.

If the hypothesis $\sum_{j=1}^{1}\lambda_{j}=1$ is added to the CRS model constraints, Equation (1) can be transformed into the VRS model, and *θ_b_* (0 < *θ_b_* ≤ 1) can be measured to denote the PTE index. The equation SE = *θ/θ_b_* can obtain the SE, where 0 < SE ≤ 1.

The key to DEA is the selection of input and output indicators. This study used the amount of land, labor, and capital inputs as input indicators and the total agricultural output value as the output indicator. Among them, land input was expressed by the actual cultivated land area owned by rural households, labor input was expressed by the total time rural households engage in agricultural production, and capital input was expressed by agricultural expenditure. Since there were many agricultural products grown by different types of rural households and it was difficult to compare the output of agricultural products, the output indicator was expressed by the total agricultural output value (Table 2).

#### 2.3.3. Tobit Regression Model

The value of CLUE calculated by DEA is a restricted dependent variable between 0 and 1, and the data is truncated, leading to bias in parameter estimation if the least-squares method is chosen. Therefore, this study used the Tobit regression model based on the maximum likelihood estimation method to analyze the effect of rural households’ CLIB on CLUE. The general expression of the Tobit regression model is as follows:

(2) $$\begin{array}{l} Y_{i}^{*}=\alpha +\beta^{\prime }X_{i}+\varepsilon_{i}\;\;\;i=1,2,3\ldots ,n\\ Y_{i}=Y_{i}^{*}\;\mathrm{if}\;Y_{i}^{*}>0\\ Y_{i}=0\;\;\;\;\mathrm{if}\;Y_{i}^{*}\leq 0 \end{array}$$

where *Y_i_* is the dependent variable characterizing the efficiency value, $Y_{i}^{*}$ is the latent variable of the dependent variable *Y_i_*, and *X_i_* is the independent variable characterizing the rural household’s input behavior. *ε_i_* is the random error term and $\varepsilon_{i}\sim N(0,\sigma )$. *i* denotes the sample ordinal number, *α* is the intercept term, and *β* is the coefficient to be estimated.

The factors influencing the CLUE are mainly labor force characteristics (age, number, and literacy level), capital characteristics (per capita household income, and share of agricultural income), cultivated land resource endowment (operation scale, effective irrigation rate, and fragmentation), technical factors (financial investment in agricultural science and technology, and guidance of agricultural technicians), and policy factors (agricultural tax exemption policy, grain cultivation subsidies) [6,19,21,40,44]. Based on the perspective of rural households, this study aims to explore the impact of rural households’ CLIB on the CLUE at the micro-level. According to the actual investigation and research purpose, 13 evaluation indicators were constructed in three aspects: labor input, capital input, and cultivated land input (Table 3).

## 3. Results

### 3.1. Statistical Analysis of Rural Household Types

**Figure 2:**
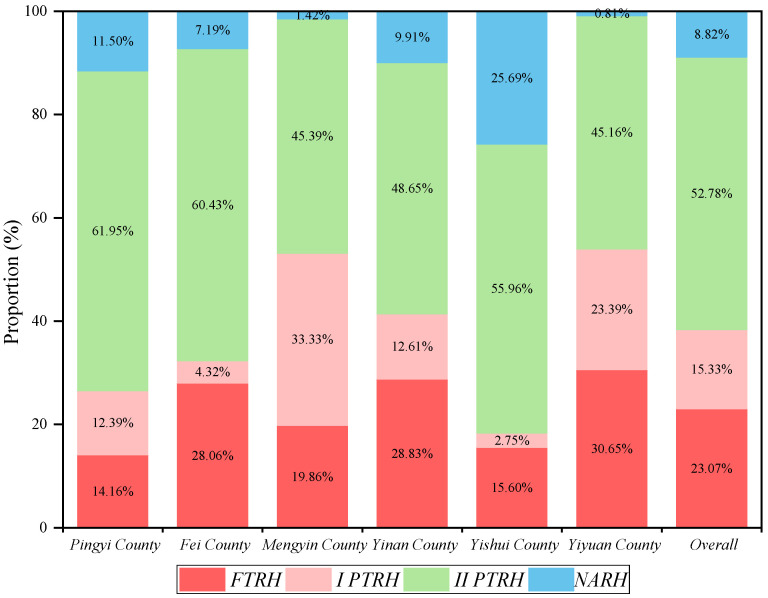
Classification of rural household types.

Part-time employment has become a common form of operation for contemporary rural households and a new way for rural households to obtain a specialized and diversified economy. In general, the number of *FTRH* was 170, accounting for 23.07% of the total sample, the number of *I PTRH* was 113, accounting for 15.33% of the total sample; the number of *II PTRH* was 389, accounting for the highest proportion of 52.78%, and the number of *NARH* was 65, accounting for the lowest percentage of 8.82%. The total number of part-time rural households exceeded 68% of the total sample, verifying the prevalence of part-time employment. From each county’s viewpoint, the highest percentage of rural household types in each county was still *II PTRH*, all exceeding 45%, with 61.95% in Pingyi County. The highest proportion of *I PTRH* was in Mengyin County, with 33.3%, the highest percentage of *NARH* was in Yishui County, with 25.69%, and the highest proportion of *FTRH* was in Yiyuan County, with 30.65% (Figure 2).

### 3.2. Characteristics of CLIB of Different Types of Rural Households

Rural households’ CLIB is closely related to their income level, which in turn affects the level of CLUE. Table 4 reflects the changes in average household cultivated land inputs for different rural household types.

#### 3.2.1. Cultivated Land Input

Regarding land input, the scale of cultivated land showed a rising and then declining trend with the increase in households’ part-time employment level. The *I PTRH* had the highest cultivated land input (4.30 mu), *FTRH* was the second-highest (3.6 mu), and *II PTRH* had less cultivated land than *I PTRH* and *FTRH*, decreasing to 3.32 mu; *NARH* had the least cultivated land, further decreasing to 1.75 mu. The number of cultivated land plots of all types of rural households exceeded 4 pieces, indicating that the fragmentation of cultivated land in the study area was relatively serious; *I PTRH* had the largest number of cultivated land plots with 7.29 pieces, and the smallest number of cultivated land plots was 4.29 pieces for *NARH*. It is easy to understand that cultivated land resources are relatively scarce in the Yimeng Mountain area, and it is difficult for households with high non-agricultural income to obtain more income through the inflow of land; thus, engaging in non-agricultural industries becomes a significant way to increase their income.

#### 3.2.2. Labor Input

Regarding labor input, *FTRH* had the most labor input per unit area of cultivated land, at 88.32 work-days/mu, and this part of rural households could still maintain intensive cultivation in production. With the improvement of non-agricultural employment, the labor input per unit area of cultivated land showed a significant decreasing trend. The labor input per unit area of cultivated land of *I PTRH* and *II PTRH* dropped to 75.67 and 60.14 work-days/mu, respectively, while that of *NARH* with the highest level of non-agricultural employment was even less, only 55.78 work-days/mu. In terms of total labor input, it showed a rising, and then declining trend as the non-agricultural employment level of rural households increased. The total labor input of *I PTRH* was the highest, 325.37 work days, followed by 317.96 work days for *FTRH*, 199.68 work days for *II PTRH,* and 97.62 work days for *NARH*. As rural households’ non-agricultural employment increases, the traditional intensive production model is being transformed, especially in the Yimeng Mountain area, which has many hills and little land.

#### 3.2.3. Capital Input

Capital inputs are the costs directly invested by rural households in the agricultural production process. Regarding capital input structure, the sum of yield-increasing inputs per unit area was much higher than labor-saving inputs for all rural household types; however, there are significant differences among the inputs.

For seed input, the average input per unit area of all rural household types was more than 78 CNY among which *I PTRH* had the highest input, reaching 204.63 CNY/mu, followed by *FTRH*. Most of these rural households used high-priced seeds, and the survey showed that 78% chose superior seed varieties. In addition, to ensure the survival rate of crops, they sowed a slightly higher than average amount of seeds per unit area of land and then eradicated those that did not survive well, thus increasing crop yields. The difference in seed inputs between *II PTRH* and *NARH* was insignificant and exceeded 100 CNY/mu. During the interviews, it was found that these rural households spent most of their time in non-agricultural employment and did not have more energy to manage agricultural production; however, they were forced to cultivate due to the pressure of abandonment, and seed input made up for the lack of their labor input.

Fertilizer, pesticide, and agricultural film inputs help to ensure crop yields. From the perspective of fertilizer inputs of different rural household types, the change rule was roughly comparable to seed inputs. The highest fertilizer input was still *I PTRH*, with 580.66 CNY/mu; *I PTRH* would achieve increased yield and income by increasing the amount of fertilizer application. The fertilizer inputs of *FTRH*, *II PTRH,* and *NARH* were 426.66, 350.6, and 285.51 CNY/mu, respectively. The highest input of pesticide and agricultural film was still *I PTRH*, reaching 297.56 and 238.46 CNY/mu, followed by *FTRH* and *NARH*, all less than 40 CNY/mu. Rural households with a higher agricultural income invest more in yield and pest control to obtain higher returns.

Mechanical power input includes activities, such as machine plowing, machine irrigation, and cutting, reflecting the extent to which farmers use machinery instead of human labor. The overall level of machinery input in the study area was low, with the highest being the *I PTRH* at only 210.86 CNY/mu. In comparison, all other types of rural households were less than 200 CNY/mu of machinery input, potentially due to cultivated land fragmentation in the study area. Compared to the cultivated land in the plains, the cultivated land in the hills and mountains is highly fragmented, making mechanized production extremely difficult [20], and rural households with smaller plots have fewer opportunities to use modern technology. *I PTRH* spent more because they owned more cultivated land and used machinery instead of human resources. The difference in mechanical power input between *FTRH* and *II PTRH* was minimal, at 152.53 and 138.6 CNY/mu, respectively; however, the mechanical power input of *NARH* was only 109.13 CNY/mu.

Regarding total capital input, *I PTRH* had the highest level of 9409.03 CNY, *FTRH* had the second-highest level of 5452.93 CNY, and *II PTRH* and *NARH* had lower levels of 3852.82 and 1117.75 CNY, respectively. Therefore, the labor and capital input levels of *I PTRH* were significantly higher than those of other types of rural households. As the level of nonfarm employment of rural households increased, they spent most of their labor time and capital on other non-agricultural activities, resulting in a decreasing trend of their labor and capital inputs in agricultural production.

### 3.3. CLUE and Distribution of Different Types of Rural Households

This study used DEAP software to measure the TE, PTE, and SE of each type of rural household in different regions. The calculated results were aggregated to obtain the average value of CLUE of overall rural households and different types of rural households to analyze the efficiency differences among different types of rural households and the spatial distribution pattern.

#### 3.3.1. Overall CLUE of Sample Rural Households

Table 5 shows that the overall average CLUE of the sample rural households in the study area was 0.19, indicating that the overall CLUE level was very low, with more space for efficiency improvement. Regarding TE composition, SE was greater than PTE, but both were lower, at 0.52 and 0.36, respectively.

#### 3.3.2. CLUE of Different Types of Rural Households

Table 5 shows the CLUE of different types of rural households. The TE of all types of rural households was low, but there were still significant differences between different types of rural households, ranked as *I PTRH* (0.32) > *FTRH* (0.18) > *II PTRH* (0.16) > *NARH* (0.05). This finding roughly shows a trend of increasing and then decreasing as the degree of households’ part-time employment increases, indicating that the appropriate part-time employment of rural households helps to improve the CLUE; however, with the gradual increase of non-agricultural employment in rural households, the CLUE showed an inverted ‘U’ shape.

Regarding PTE, all types of rural households were generally low, with the highest *NARH* being only 0.46. Concerning SE, *I PTRH* was the highest, followed by *FTRH* and *II PTRH*, and *NARH* was the lowest. For the overall sample of rural households, SE was higher than PTE, indicating that different types of rural households had different production and operation methods; the difference in SE was the key to variations in CLUE. From the scale payoff viewpoint, most rural households were in the stage of increasing returns to scale, which also indicated that the scale of agricultural production of different rural household types in the study area was generally small, consistent with the characteristics of the smallholder management mode.

#### 3.3.3. Distribution of CLUE within Different Types of Rural Households

**Figure 3:**
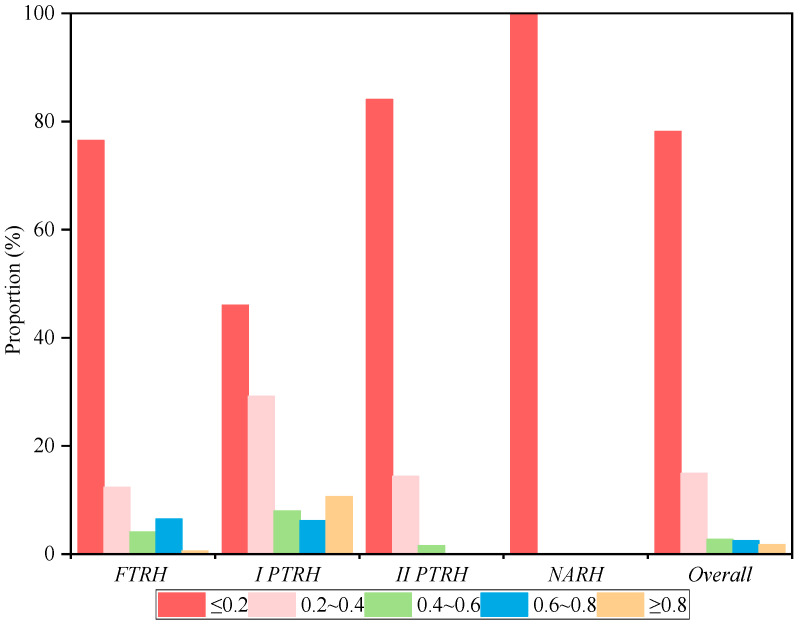
Distribution of CLUE within different types of rural households.

Statistical analysis was conducted on the distribution of CLUE for different types of rural households (Figure 3) to explore the distribution characteristics of CLUE in each type of rural household. Based on the current research results [2,42], this study chose 0.8 as the cut-off point, and those with efficiency values above 0.8 were considered high efficiency. Among the sample rural households, 78.15% had efficiency below 0.2, 14.93% had efficiency between 0.2 and 0.4, 2.71% had efficiency between 0.4 and 0.6, 2.44% had efficiency between 0.6 and 0.8, and only 1.76% of the rural households had high efficiency. The distribution characteristics of CLUE of different rural household types were similar; the highest percentage of rural households with an efficiency below 0.2 was more than 46% among which all *NARH* had an efficiency below 0.2. The highest proportion of high efficiency was for *I PTRH*, 10.62%, while the percentage of *II PTRH* and *NARH* was 0. This result also indicated that appropriate part-time employment was conducive to improving CLUE, while CLUE would decline when the degree of part-time employment was relatively high. The CLUE of different rural household types was unevenly distributed and generally low, closely related to the difference in CLIB. Therefore, it is necessary to investigate the impact of CLIB on CLUE further.

### 3.4. Effects of CLIB on CLUE of Different Types of Rural Households

The 13 indicators affecting CLUE were quantified as explanatory variables, and the CLUE (TE) was quantified as the explained variables. Due to the large number of variables selected, the possibility of multicollinearity existed; therefore, multicollinearity tests were performed before model estimation. In this study, the variance inflation factor (VIF) was used as the basis for judging the multicollinearity test; if the variance inflation factor VIF > 10, serious multicollinearity exists among the variables, and this variable can be removed so that other suitable explanatory variables can be selected. Table 6 shows the results of multicollinearity diagnosis of explanatory variables for different types of rural households. The VIF of 13 explanatory variables for each type of rural household was less than 10, indicating no serious multicollinearity among explanatory variables, and the variables could be retained.

On this basis, the Tobit regression model analysis was conducted using STATA software to analyze the factors influencing the CLUE for different types of rural households. The significance tests were conducted on the regression coefficients (Table 7). According to the regression model results, Log likelihood values were 282.712, 293.058, 363.820 and 255.338, respectively; Wald chi2 all passed the 1% significance level test, indicating that the Tobit model was significant.

#### 3.4.1. The Impact of CLIB on CLUE of *FTRH*

Among the explanatory variables of labor force input characteristics of *FTRH*, the literacy level of agricultural labor force had a significant positive effect on CLUE, indicating that the higher the labor force’s literacy level, the more conducive it is to improving CLUE. Among the explanatory variables of capital input characteristics, yield-increasing input per unit area, per capita household income, and share of agricultural income significantly and positively affected CLUE. This result suggests that enhancing the yield-increasing input and increasing the level of rural households’ income were conducive to improving CLUE; however, the proportion of agricultural capital input had a significant negative effect on CLUE, indicating that the higher the proportion of agricultural capital input, the less favorable it is to improving the CLUE of *FTRH*. Among the explanatory variables of land input characteristics, the operation scale of cultivated land and the effective irrigation rate of cultivated land both had significant positive effects on CLUE, reflecting the essential roles of cultivated land resource endowment and agricultural infrastructure on CLUE.

#### 3.4.2. The Impact of CLIB on CLUE of *I PTRH*

Among the explanatory variables of labor input characteristics of *I PTRH*, the amount of agricultural labor force and their literacy level had significant positive effects on CLUE, indicating that the higher the agricultural labor force in the household, the higher the literacy level contributed to CLUE. Among the explanatory variable of capital input characteristics, yield-increasing inputs per unit area, per capita household income, and share of agricultural income all significantly and positively affected CLUE; however, the labor-saving inputs per unit area had a significant negative impact on CLUE. The topography of the study area mainly constrained labor-saving inputs, as the fragmentation of plots was more serious and challenging to mechanize, thus limiting the improvement of CLUE. Among the explanatory variables of land input characteristics, the soil and water conservation rate of cultivated land had a significant positive effect on CLUE, indicating that implementing soil and water conservation measures by rural households reduced the occurrence of soil erosion to a certain extent, which protected land resources and productivity and helped to improve CLUE.

#### 3.4.3. The Impact of CLIB on CLUE of *II PTRH*

Among the explanatory variables of capital input characteristics of *II PTRH*, the proportion of agricultural capital input had a significant negative effect on CLUE. This impact was mainly because agricultural production was less profitable for *II PTRH*, leading to insufficient incentive to invest in agricultural capital and reducing capital input accordingly. The significant positive effect of yield-increasing inputs per unit area on CLUE again indicated that enhancing yield-increasing inputs per unit area could effectively improve CLUE; however, in terms of labor input and land input characteristics, the variables did not significantly affect CLUE, but the direction of influence was basically the same as that of *FTRH* and *I PTRH*.

#### 3.4.4. The Impact of CLIB on CLUE of *NARH*

Among the explanatory variables of labor input characteristics of *NARH*, the amount of agricultural labor force had a significant negative impact on CLUE, differing significantly from that of *FTRH*, *I PTRH,* and *II PTRH*. The main reason was that *NARH* had insufficient agricultural labor and was mainly allocated to non-agricultural activities; the lack of labor force and poor management led to a lower CLUE. Among the explanatory variables of capital input characteristics, both per capita household income and share of agricultural income had a significant positive effect on CLUE; however, the proportion of agricultural capital input had a significant negative impact on CLUE. Among the explanatory variables of land input characteristics, cultivated land fragmentation degree had a significant negative effect on CLUE, mainly because the higher the degree of cultivated land fragmentation, the greater the number of plots and the more unfavorable to centralized and continuous management of cultivated land. These factors hindered the use of large agricultural machinery, thus increasing the land input costs of rural households.

In summary, the influence of different types of rural households’ CLIB on CLUE was basically in the same direction; however, there was significant heterogeneity in the degree of influence.

## 4. Discussion

### 4.1. CLIB of Different Types of Rural Households

This study analyzed the CLIB of rural households, and the results reflected heterogeneity among different types of rural households. *I PTRH* was significantly higher than other types of rural households in terms of land, labor, and capital inputs. This was mainly because, on the one hand, the household size of *I PTRH* and the total area of cultivated land they owned were larger, so the total input increased accordingly. The survey indicated that 34.5% of the sample households of this type had rented in cultivated land, and only 6.19% had rented out cultivated land, resulting in a larger scale of their operating cultivated land. On the other hand, the agricultural income of *I PTRH* was the primary source of income for their households. Most rural households grew economic crops with complicated daily management and a high labor demand, such as peaches, apples, and cherries. Furthermore, some family members went out to work to increase income, relaxing the household’s budgetary constraints on agricultural production. Rural households prioritized acquiring productive agricultural assets to increase land output, leading to high labor and capital inputs. The extant literature supports this view, suggesting that the increased income and easing of mobility constraints brought by family members working outside the home helped to promote investment in agricultural production and that working outside the home motivated rural households to purchase more agricultural machinery and fertilizers. Additionally, they tended to use their non-agricultural income for investment in agricultural production, which was consistent with the research of Chiodi et al. [45], De Brauw [46], Taylo and Lopez-Feldman [47] and Zhao [48]. These studies suggested that appropriate part-time employment of rural households would increase agricultural investment capacity and risk resistance, especially the increased purchasing power of agricultural machinery and fertilizer.

In contrast, *II PTRH* with higher levels of non-agricultural employment was lower in cultivated land inputs than the *FTRH* and *I PTRH*. The household income source of *II PTRH* was mainly non-agricultural, with the average share of non-agricultural income reaching 80.3%. Nonetheless, *II PTRH* did not increase its investment in agricultural production due to the non-agricultural employment of family members. The extant literature supports this view that non-agricultural employment reduced the input of labor, capital, and other factors per unit of land, causing a change in the original intensive farming pattern [49,50]. On the one hand, this result may arise from non-agricultural income being mainly used for non-productive expenditures, such as improving the livelihood of rural households and purchasing durable goods [51]. On the other hand, it may be that non-agricultural income was used for other types of investments, such as housing and education investments. Similar results were found by Quisumbing and Mcniven [52] and Zhu et al. [53].

*NARH* had very low levels of land, labor, and capital inputs, mainly because, on the one hand, most *NARH* had a “two-generation” family structure, requiring them to care for young children, educate school-age youth, or support the elderly. Therefore, the labor input in agricultural production was minimal. On the other hand, *NARH* had a relatively young labor force with a relatively high level of education. They were usually engaged in non-agricultural employment rather than agricultural production, where local non-agricultural employment provided a higher household income, and agricultural production was considered only basic food security. The survey found that the vast majority of *NARH* chose to grow wheat, maize, and other food crops that were easy to manage and had less investment, resulting in less input in their labor and capital. Some researchers had a similar view, arguing that rural households working outside the home for a long time shifted their employment focus and even possessed the possibility of off-farm production, reducing investment in agricultural production [54,55]. More specifically, it induced a shift of young laborers with higher education and skills to non-agricultural production, reducing the input to agricultural production [56]. These studies all indicated that as the degree of part-time employment deepened, the dependence of rural households on agriculture would gradually decrease. Most rural households did not expect to increase their income through agriculture, so the agricultural production and operation methods gradually became extensive. At this time, rational rural households prioritize non-agricultural industries with higher returns as investment areas, and their investment in agriculture may decrease.

### 4.2. CLUE and Distribution of Different Types of Rural Households

The CLUE analysis in rural households showed that the CLUE in the study area was minimal and had considerable potential for improvement. The reasons for this were, on the one hand, that the topography of the study area was mainly mountainous and hilly, and the terrain was more fragmented, resulting in poor agricultural production conditions for cultivated land resources. On the other hand, the economic development of the study area was relatively slow. In contrast, with the development of secondary and tertiary industries, the opportunity cost of agricultural labor was rising, and many young and strong rural laborers were transferred to non-agricultural industries. This transfer resulted in the decline in the quantity and quality of rural labor, leading to a low CLUE under both nature and socio-economic influences.

The CLUE of different types of rural households also differed significantly. The highest CLUE was found in the *I PTRH*, while the lowest was in the *II PTRH* and *NARH*; namely, the CLUE showed an inverted ‘U’ shaped trend as the non-agricultural employment level of rural households gradually increased. Similar results were found by Xu and Chen [42] and Yang et al. [57], namely, there was a robust inverted ‘U’ shaped relationship between the level of non-agricultural employment of rural households and CLUE. This phenomenon was related to rural households’ business objectives and input land, labor, and capital choices. With maximization of agricultural returns as the primary goal, *I PTRH* tended to use the non-agricultural household income for agricultural production investment, alleviating household mobility and capital constraints and improving CLUE [58]. In contrast, *II PTRH* and *NARH* took non-agricultural activities as the primary livelihood strategy of the household. They tended to use the non-agricultural household income for non-productive investment, leading to a decrease in the input of labor, capital, and other factors per unit of land, which was not conducive to improving CLUE. The finding was consistent with the research of Damon [59].

Regarding PTE, all types of rural households were generally low, indicating that, on the one hand, the mastery and application of agricultural production skills of the sample rural households in the study area were low. On the other hand, it indicated that the update rate of agricultural production technology was relatively slow, and the agricultural technology promotion system needed to be improved. It was noteworthy that *NARH* had the highest PTE but the lowest TE and SE. This finding indicated that rural households’ knowledge, information, and income from participation in non-agricultural activities helped to improve their agricultural productivity, such as applying additional farmyard manure, soil testing, and fertilization. Rural households were also encouraged to participate in fallow and crop rotation programs, thus improving the PTE of agricultural production. Similar results were found by Wouterse et al. [60].

### 4.3. Effects of CLIB on CLUE of Different Types of Rural Households

There were similarities and heterogeneity in the effects of different types of rural households’ CLIB on CLUE. Regarding labor input characteristics, the literacy level of agricultural labor had a significant positive effect on both *FTRH* and *I PTRH*, indicating that laborers with higher literacy could learn technologies and knowledge related to agricultural production and had a stronger ability to accept and master new technologies. At the same time, they could make timely adjustments in the face of market demand, thus contributing to improving CLUE. The finding was consistent with the research of Xia et al. [61]. The amount of agricultural labor force had a significant positive effect on *I PTRH* and a significant negative impact on *NARH*, mainly because *I PTRH* took agricultural income as their primary source of income. Additionally, the more labor force available in rural households for agricultural production, the more likely they were to increase their investment in cultivated land, thus further increasing agricultural output. Conversely, *NARH* took non-agricultural income as its primary source of income and was more willing to invest labor in non-agricultural activities. In other words, non-agricultural employment had a “labor loss effect” on agricultural production; namely, it led to neglect of production and reduced household agricultural labor inputs. Zhao et al. [6] took 1961 counties in China as the research objects, and also obtained the same results.

Regarding capital input characteristics, the yield-increasing inputs per unit area had significant effects on *FTRH*, *I PTRH* and *II PTRH*, indicating that rural households could increase the output per unit area of cultivated land by applying good seeds, fertilizers, and pesticides, thus improving CLUE. Since *NARH* had only a small amount of cultivated land for food rations or elderly cultivation and required a limited amount of production materials, such as pesticides, fertilizers, and seeds, the CLUE did not increase significantly with rising yield-increasing inputs. Meanwhile, per capita household income and share of agricultural income also had significant effects on *FTRH*, *I PTRH,* and *II PTRH*. This finding reflects that higher per capita and agricultural income could increase rural households’ motivation to engage in agricultural production and have sufficient funds available to improve agricultural production techniques and facilities, which could improve CLUE. Notably, the proportion of agricultural capital inputs significantly and negatively affected *FTRH*, *I PTRH,* and *II PTRH*, indicating that more agricultural capital inputs were not better. Furthermore, excessive inputs could cause substantial redundancy, which was not conducive to improving CLUE. Similar results were found by Ge et al. [62], Wu et al. [63] and Ito [64] in which they also noted that excessive capital inputs inhibited CLUE. However, the negative effect of labor-saving inputs per unit area was only significant for *I PTRH*, not for other types of rural households. This difference is mainly because *I PTRH* had the most cultivated land plots and the most severe cultivated land fragmentation, hindering the performance of labor-saving inputs and limiting CLUE.

Regarding land input characteristics, the operation scale of cultivated land and the effective irrigation rate of cultivated land only had significant effects on *FTRH* and positive but insignificant effects on other types of rural households. This result indicated that cultivated land had a typical payoff effect of scale; however, there should be an appropriate range of cultivated land operation, not the larger, the better. Too large an operation scale of cultivated land for rural households could lead to inefficient use of cultivated land and a mismatch with labor, capital, technology, and other factors. The finding was consistent with the research of Ferreira et al. [14] and Chen et al. [65]. Conversely, it also showed that improving agricultural infrastructure and irrigation conditions improved CLUE. Some studies indicated that investment in water conservancy facilities for cultivated land was one main factor affecting CLUE [66]; however, the cultivated land fragmentation degree had a significant negative effect on *FTRH* and a negative but non-significant effect on other types of rural households. This finding indicated that the cultivated land fragmentation prevented capital and labor inputs from generating scale effects, increased the production costs of rural households, and was not conducive to improving CLUE. Zhou et al. [67] and Rahman [68] also found similar results, namely, cultivated land fragmentation significantly reduced CLUE. The soil and water conservation rate of cultivated land only had a significant positive effect on *I PTRH*; the effect on other types of rural households was positive but not significant. This difference was because, on the one hand, *I PTRH* valued agricultural production and was willing to invest in soil and water conservation construction of cultivated land. On the other hand, part-time employment income relieved financial constraints, thus enabling *I PTRH* to invest in soil and water conservation. The finding was consistent with the research of Eder et al. [69] in which he stated that a substantial improvement potential existed in the soil conservation behavior of farms.

The above explanatory variables that significantly affected CLUE were those derived from the Tobit regression analysis results. Regarding other influencing factors, the age of agricultural labor and the transfer of cultivated land negatively affected CLUE but did not pass the significance test; however, this did not mean that they did not affect CLUE. For example, the age of the agricultural labor force negatively affected CLUE because agricultural production required a certain amount of physical and energy input. In general, older people in agricultural production have higher productivity than younger people. Yang et al. [57] also pointed out in their study that compared with the productivity of younger adults, the productivity of older adults in agricultural production was lower. In contrast, the agricultural labor force in the study area was more severely aged and had a reduced labor capacity, which to a certain extent limits the improvement of CLUE.

### 4.4. Policy Implications

With the development of the social economy, livelihood strategies and income structures of rural households gradually differentiate, creating variances in CLIB and CLUE of different types of rural households. This is prone to irrational utilization of production factors, such as land, labor, and capital, threatening national arable land security and food security. Solving this problem requires guiding the coordinated development of rural households’ input decisions and cultivated land utilization. To this end, improvements can be made from the perspectives of the different rural household types. The following are some suggestions.

Differentiated cultivated land input optimization plans can be developed according to different rural household types. (1) *FTRH* can enrich its agricultural knowledge reserves and improve its professional quality by organizing collective learning. At the same time, it is necessary to improve agricultural infrastructure, moderately expand the planting scale, and guide *FTRH* to grow higher value-added agricultural products according to local characteristics to improve agricultural returns. (2) *I PTRH*, on the one hand, can optimize the structure of agricultural production, strengthen the construction of soil and water conservation infrastructure, increase the promotion of agricultural technology, and improve their specialization in seed selection and fertilization. On the other hand, *I PTRH* can enrich the scope of non-agricultural activities to increase its income, choose more appropriate and efficient non-agricultural activities, and coordinate the relationship between agricultural and non-agricultural activities. (3) For *II PTRH*, on the one hand, the land-use structure can be flexibly adjusted according to the working hours and work content, and the household labor force and capital can be reasonably allocated. On the other hand, *II PTRH* can guarantee its enjoyment of property rights to land and rental income, promoting the transfer of cultivated land resources to highly productive farmers and improving the efficiency of labor resources and CLUE. (4) For *NARH*, on the one hand, the government can provide diversified employment channels and create more non-agricultural employment opportunities, while providing skills training to enhance non-agricultural competitiveness. On the other hand, the government can encourage *NARH* to engage in land transfer, land leasing, and land-sharing to give up land management rights to work in cities without worries. In addition, *NARH* can join rural production cooperatives and use cultivated land resources to receive certain income dividends.

### 4.5. Limitations

Although our study provides insights into the characteristics of cultivated land inputs of different types of rural households in the Yimeng Mountain area and their effects on the heterogeneity of CLUE, several limitations still need to be addressed. First, this study focused on the quantitative characterization of CLIB in different types of rural households. Therefore, only direct indicators characterizing land, capital, and labor were selected, without considering indirect indicators, such as cultivated land quality, labor structure, and crop planting structure. Second, in analyzing the factors influencing CLUE, due to the limitations of data collection, this study only considered the factors of CLIB that affected CLUE. It did not consider the role of invisible factors, such as natural conditions, economic development level, or other policy variables, so the factors indirectly affecting CLUE need further exploration in future research. The behavior of cultivated land inputs of rural households can not only affect CLUE, but also may produce a series of environmental pollution problems. Therefore, it is necessary to strengthen the research on the impact of cultivated land input on ecological environment in the future. In addition, the results of this study relied on the field survey data from rural households in the Yimeng Mountain area of northern China. This study would be more enlightening with data from other parts of China for comparative analysis.

## 5. Conclusions

Based on the field survey data of 737 rural households in the Yimeng Mountain area, this study systematically analyzed the characteristics of CLIB of different types of rural households, measured their CLUE using the DEA model, and explored the influence of CLIB on CLUE based on the Tobit regression model. Compared to other countries or regions with similar problems, the contributions and differences of this study focused on the following two aspects. First, it systematically examined the input characteristics of different types of rural households in the Yimeng Mountain area in terms of land, capital, and labor and the heterogeneity of CLUE. The findings could provide empirical support for coordinating the relationship between rural households and cultivated land utilization and promoting agricultural development and revitalization in rural households’ part-time employment in China. Second, it focused on the interrelationship and influence between CLIB and CLUE, exploring the relationship between the two from the perspective of rural households’ part-time employment, enriching and expanding the CLUE research. The main findings are as follows.

(1) The characteristics of CLIB of different types of rural households differed significantly. Regarding land input, *I PTRH* had the highest, 4.30 mu. Regarding labor input, *FTRH* had the highest labor input per unit area, 88.32 work-days/mu, and *I PTRH* had the highest total labor input, 325.37 work-days. Regarding capital input composition, the sum of yield-increasing input per unit area was much higher than labor-saving input for all types of rural households. *I PTRH* had the highest level in total capital input, at 9409.03 CNY, and the highest land input, labor input, and capital input. In contrast, *II PTRH* and *NARH* had higher non-agricultural employment, but the input levels gradually decreased, indicating that appropriate part-time employment would promote rural households to increase the effective input of cultivated land. When the degree of part-time employment exceeds a certain threshold, it may decrease cultivated land input;
(2) The CLUE of the sample rural households in the study area was generally low at 0.19, with considerable potential for efficiency improvement. There were still significant differences in CLUE for different types of rural households. As the degree of part-time employment in rural households increased, CLUE showed an inverted U-shaped trend, first increasing and then decreasing, namely, *I PTRH* > *FTRH* > *II PTRH* > *NARH*, indicating that appropriate part-time employment in rural households could help to improve CLUE. From the composition of CLUE, the difference in SE was the crucial reason for the difference in CLUE for each rural household type. Most rural households were in the stage of increasing scale returns, indicating that the scale of agricultural production of rural households in the study area was generally small;
(3) The influence of CLIB of different rural household types on CLUE was basically in the same direction; however, there was significant heterogeneity in the degree of influence. The literacy level of the agricultural labor force, yield-increasing input per unit area, per capita household income, the share of agricultural income, operation scale of cultivated land, effective irrigation rate of cultivated land, and soil and water conservation rate of cultivated land tended to affect CLUE of rural households positively. In contrast, the proportion of agricultural capital input, labor-saving input per unit area, and cultivated land fragmentation degree tended to affect rural households’ CLUE negatively. Although the influences of the age of the agricultural labor force and cultivated land transfer were insignificant, this does not mean that they were unrelated to CLUE.

## Figures and Tables

**Table 1:** Criteria for classifying rural household types [43].

Types of Rural Households	Main Livelihood	Allocation Method of Agricultural and Sideline Products	Main Sources of Household Income	Household Income Structure
*FTRH*	Agriculture	Mostly for market allocation, a few for self-production and self-sales	Agricultural income, government subsidies	*AIP* > 95%
*I PTRH*	Agriculture, non-agriculture	Mostly for market allocation, a few for self-production and self-sales	Agricultural income, non-agricultural income	50% < *AIP* ≤ 95%
*II PTRH*	Non-agriculture, agriculture	Partly for market allocation, a few for self-production and self-sales	Non-agricultural income, agricultural income	50% < *NAIP* ≤ 95%
*NARH*	Non-agriculture	Mostly for self-production and self-sales, a few for market allocation	Non-agricultural income	*NAIP* > 95%

Note: *AIP* represents the proportion of agricultural income in total household income; *NAIP* represents the proportion of non-agricultural income in total household income.

**Table 2:** Input–output indicators for rural households’ CLUE evaluation.

Indicator Type	Input Indicator			Output Indicator
	*I*_1_	*I*_2_	*I*_3_	*O*_1_
Variables	Cultivated land area	Labor input	Agricultural expenditure	Total agricultural output value
Units	mu	work-day	CNY	CNY

Note: Mu is a unit of land area in China, 1 mu = 0.0667 ha; the mean conversion rate for the year covered by the survey is 6.6 CNY: 1 USD.

**Table 3:** Evaluation indicators of factors influencing the CLUE.

Category	Variables	Variable Assignment and Description
Labor input	Age of agricultural labor force (*X*_1_)	The average age of household agricultural labor force (year)
	Amount of agricultural labor force(*X*_2_)	The number of the household agricultural labor force (person)
	Literacy level of agricultural labor force (*X*_3_)	The average education level of household agricultural labor (illiteracy = 1; primary school = 2; junior high school = 3; high school = 4; college or higher = 5)
Capital input	Proportion of agricultural capital input (*X*_4_)	The agricultural capital inputs as a share of total household income (%)
	Yield-increasing input per unit area (*X*_5_)	The sum of the cost of seeds, pesticides, fertilizers, and agricultural film per unit area (CNY/mu)
	Labor-saving input per unit area (*X*_6_)	The cost of agricultural machinery power per unit area (CNY/mu)
	Per capita household income (*X*_7_)	Ratio of total household income to total population (CNY/person)
	Share of agricultural income (*X*_8_)	The share of agricultural income in total household income (%)
Cultivated land input	Operation scale of cultivated land (*X*_9_)	The total area of cultivated land actually put into production by households (mu)
	Cultivated land fragmentation degree (*X*_10_)	The number of plots of cultivated land for households (piece)
	Effective irrigation rate of cultivated land (*X*_11_)	The effective irrigated cultivated land area of households as a proportion of total cultivated land area (%)
	Soil and water conservation rate of cultivated land (*X*_12_)	The proportion of cultivated land area where households take soil and water conservation measures to total cultivated land area (%)
	Cultivated land transfer status (*X*_13_)	Transfer in = 1; Not involved in the transfer = 0; Transfer out = −1

**Table 4:** Inputs of different types of rural households.

Types of Rural Households	Cultivated Land Input		Labor Input		Yield-Increasing Input				Labor-Saving Input	Total Capital Input (CNY)
	Cultivated Land Area (mu)	Number of Cultivated Land Plots (piece)	Labor Input per Unit Area (work-day/mu)	Total Labor Input (work-day)	Seed (CNY/mu)	Pesticide (CNY/mu)	Fertilizer (CNY/mu)	Agricultural Film (CNY/mu)	Mechanical Power (CNY/mu)	
*FTRH*	3.60	5.15	88.32	317.96	188.10	197.76	426.66	48.09	152.53	5452.93
*I PTRH*	4.30	7.29	75.67	325.37	204.63	297.56	580.66	238.46	210.86	9409.03
*II PTRH*	3.32	5.49	60.14	199.68	155.12	108.15	350.60	23.50	138.60	3852.82
*NARH*	1.75	4.29	55.78	97.62	113.26	39.80	285.51	25.17	109.13	1117.75

**Table 5:** CLUE and scale payoff for different rural household types.

Types of Rural Households	TE	PTE	SE	Increasing Returns to Scale/%	Decreasing Returns to Scale/%	Constant Returns to Scale/%
*FTRH*	0.18	0.36	0.46	98.82	0.59	0.59
*I PTRH*	0.32	0.40	0.73	93.04	3.48	3.48
*II PTRH*	0.16	0.38	0.42	100.00	-	-
*NARH*	0.05	0.46	0.13	100.00	-	-
Overall	0.19	0.36	0.52	98.64	0.68	0.68

**Table 6:** The multicollinearity diagnosis results of explanatory variables for different types of rural households.

Explanatory Variables	*FTRH*	*I PTRH*	*II PTRH*	*NARH*
	VIF	VIF	VIF	VIF
Age of agricultural labor force (*X*_1_)	1.90	1.56	1.14	1.68
Amount of agricultural labor force(*X*_2_)	1.34	1.30	1.04	1.25
Literacy level of agricultural labor force (*X*_3_)	1.33	1.47	1.10	1.89
Proportion of agricultural capital input (*X*_4_)	2.39	2.89	3.23	5.51
Yield-increasing input per unit area (*X*_5_)	1.87	1.97	1.97	4.38
Labor-saving input per unit area (*X*_6_)	1.78	1.49	1.62	5.09
Per capita household income (*X*_7_)	1.92	2.18	1.25	1.30
Share of agricultural income (*X*_8_)	1.92	1.31	2.02	3.94
Operation scale of cultivated land (*X*_9_)	1.75	1.83	1.81	2.30
Cultivated land fragmentation degree (*X*_10_)	1.19	1.37	1.29	2.39
Effective irrigation rate of cultivated land (*X*_11_)	1.15	1.45	1.13	5.09
Soil and water conservation rate of cultivated land (*X*_12_)	1.75	1.57	1.14	2.34
Cultivated land transfer status (*X*_13_)	1.38	1.28	1.31	1.12

**Table 7:** The Tobit regression results of CLUE of different types of rural households.

Explanatory Variables	*FTRH*		*I PTRH*		*II PTRH*		*NARH*	
	Coefficient	T-Value	Coefficient	T-Value	Coefficient	T-Value	Coefficient	T-Value
Age of agricultural labor force (*X*_1_)	−0.230	−1.36	−0.269	0.78	−0.034	−0.37	−0.279	−1.68
Amount of agricultural labor force(*X*_2_)	0.062	0.75	0.131 *	0.78	0.028	0.44	−0.299 **	−2.14
Literacy level of agricultural labor force (*X*_3_)	0.183 ***	3.88	0.206 **	2.01	0.017	1.21	0.026	0.27
Proportion of agricultural capital input (*X*_4_)	−0.521 ***	−11.49	−0.079	−0.95	−0.062 ***	−2.64	−0.434 ***	−4.97
Yield-increasing input per unit area (*X*_5_)	0.042 *	1.91	0.186 ***	2.85	0.036 **	1.98	0.038	0.44
Labor-saving input per unit area (*X*_6_)	0.026	0.75	−0.126 *	−1.880	0.005	0.32	−0.044	−0.86
Per capita household income (*X*_7_)	0.063 **	2.08	0.130 *	1.19	0.052	1.68	0.130 *	1.68
Share of agricultural income (*X*_8_)	0.600 ***	8.69	0.123 *	0.58	0.042	1.68	0.662 ***	6.52
Operation scale of cultivated land (*X*_9_)	0.076 ***	2.89	0.094	0.56	0.049	1.30	0.016	0.24
Cultivated land fragmentation degree (*X*_10_)	−0.018	−0.48	−0.087	−1.35	−0.033	−1.04	−0.131 *	−1.84
Effective irrigation rate of cultivated land (*X*_11_)	0.075 ***	3.24	0.101	−1.15	0.028	1.31	0.015	0.91
Soil and water conservation rate of cultivated land (*X*_12_)	0.002	0.19	0.050 *	1.74	0.006	0.94	0.004	0.65
Cultivated land transfer status (*X*_13_)	−0.004	−1.57	−0.006	−0.39	−0.001	−0.40	−0.046	−0.82
Constant	0.423	1.63	0.237	0.44	0.912	6.93	0.823	3.20
sigma_u	0.135 ***	12.35	0.115 ***	10.57	0.145 ***	14.30	0.141 ***	12.72
sigma_e	0.105 ***	25.49	0.139 ***	25.14	0.128 ***	25.22	0.105 ***	25.63
Log likelihood	282.712	0.000	293.058	0.000	363.820	0.000	255.338	0.000
Wald chi2(13)	95.82	737	119.31	737	127.74	737	80.92	737

Note: *, **, and *** indicate significant levels at 10%, 5%, and 1% levels, respectively.

## Data Availability

The data used to support the findings of this study are available from the corresponding author upon request.
